# 

**Published:** 1908-01

**Authors:** 


PUBLISHED MONTHLY. ATLANTA SUBSCRIPTION $1.00

### JOURNAL-RECORD OF MEDICINE

Successor to { Atlanta Medical and Surgical Journal, Established 1855. and Southern Medical Record, Established 1870. } 52d Year

Vol. IX. ATLANTA, GA., JANUARY, 1908. No. 10

